# Endothelial IRE1 signaling maintains blood–brain barrier integrity and limits neuroinflammation after traumatic brain injury

**DOI:** 10.1038/s41419-026-08461-2

**Published:** 2026-02-09

**Authors:** Qiyan Fan, Mika Takarada-Iemata, Takashi Tanaka, Loc Dinh Nguyen, Nahoko Okitani, RongRong Yang, Takashi Tamatani, Hiroshi Ishii, Tsuyoshi Hattori, Hiroyasu Kidoya, Yoshiaki Kubota, Takao Iwawaki, Osamu Hori

**Affiliations:** 1 Department of Neuroanatomy, Graduate School of Medical Sciences, Kanazawa University, Ishikawa, Japan; 2 Department of Rehabilitation Science, Graduate School of Health Sciences, Kobe University, Hyogo, Japan; 3 Department of Integrative Vascular Biology, Faculty of Medical Science, Fukui University, Fukui, Japan; 4 Department of Anatomy, Keio University School of Medicine, Tokyo, Japan; 5 Division of Cell Medicine, Department of Life Science, Medical Research Institute, Kanazawa Medical University, Ishikawa, Japan

**Keywords:** Blood-brain barrier, Diseases of the nervous system, Stress signalling, Cell adhesion, Extracellular signalling molecules

## Abstract

Endoplasmic reticulum (ER) stress and activation of the unfolded protein response (UPR) contribute to the pathogenesis of traumatic brain injury (TBI), yet the cell type-specific roles of UPR pathways remain poorly understood. We previously identified endothelial cells (ECs) as a primary site of IRE1 pathway activation following brain injury. In this study, we investigated the role of endothelial IRE1 signaling in TBI using EC-specific IRE1 conditional knockout mice subjected to cortical ablation. Loss of IRE1 in ECs exacerbated blood-brain barrier (BBB) disruption, enhanced immune cell infiltration, amplified neuroinflammation, and expanded neuronal damage, ultimately leading to worsened neurological outcomes. RNA-sequencing revealed enrichment of interferon-related programs and identified Cxcl10 as an endothelial chemokine linked to the exacerbated leukocyte recruitment in endothelial IRE1 deficiency. Treatment with the chemical chaperone tauroursodeoxycholic acid (TUDCA) suppressed Cxcl10 expression both in vitro and in vivo, and significantly improved motor function following TBI. These findings reveal a critical role for endothelial IRE1 signaling in maintaining BBB integrity and restraining inflammation during the acute phase of TBI. Modulation of ER stress in brain ECs may represent a promising and accessible therapeutic strategy for reducing secondary injury after TBI.

## Introduction

Traumatic brain injury (TBI) is a leading cause of death and disability worldwide, and a common form of acute injury to the central nervous system (CNS) [1]. In general, the pathophysiology of TBI is categorized into two phases: primary injury, involving immediate structural damage to brain tissue, and secondary injury, comprising a cascade of molecular and cellular processes that progress over hours to weeks after the initial trauma. These secondary injury processes, which include excitotoxicity, oxidative stress, blood-brain barrier (BBB) disruption, peripheral immune cell infiltration, and neuroinflammation, interfere with tissue repair and impede functional restoration [2]. Thus, elucidating the molecular mechanisms underlying secondary injury is essential for developing effective therapeutic strategies.

Among the processes mentioned above, BBB disruption stands out as a pivotal event following TBI [3, 4]. Mechanical stress on the cerebral microvasculature increases BBB permeability, accompanied by downregulation of tight junction proteins and loss of barrier integrity. As a result, the brain parenchyma is exposed to inflammatory mediators and cytotoxic factors, all of which trigger immune cell infiltration and amplify the inflammatory cascade. These events play a key role in the development of secondary injury after TBI, and are therefore tightly regulated by intracellular stress responses.

Among various intracellular pathways, endoplasmic reticulum (ER) stress and the unfolded protein response (UPR) are recognized widely as central regulators of CNS pathology [5]. Cells experiencing ER stress activate the UPR to restore ER homeostasis and maintain proper protein folding capacity [6]. The UPR consists of three canonical signaling branches, among which the inositol-requiring enzyme 1 (IRE1) pathway is the most evolutionarily conserved. IRE1 signaling is activated in CNS disorders such as spinal cord injury, Alzheimer’s disease, and multiple sclerosis, and is associated with neuronal cell death, glial activation, neuroinflammation, and cognitive dysfunction [7–9]. While numerous studies have used pharmacological agents and conditional knockout (cKO) models targeting neurons and glia to elucidate the functions of UPR, the cell type-specific roles played by individual UPR pathways across the neurovascular unit during disease progression remain unclear.

Our previous study using acute brain injury models and ER-stress-activated indicator (ERAI) transgenic mice, which allows in vivo visualization of IRE1 activation [10], identified endothelial cells (ECs) as the predominant cell population exhibiting IRE1 pathway activation following injury [11]. These findings suggest that the IRE1 pathway in brain ECs may functionally contribute to post-traumatic pathology. Although IRE1 signaling is implicated in placental development [12], angiogenesis during retinal development, and hindlimb ischemia [13], its role in brain ECs under pathological conditions remains largely unexplored.

In this study, we generated EC-specific IRE1 cKO mice and subjected the mice to a cortical ablation TBI model to investigate the contribution of endothelial IRE1 signaling to post-injury pathology. Through behavioral assessment, immunohistochemistry, and molecular analyses, we demonstrate that endothelial IRE1 attenuates neurological deficits after TBI by preserving BBB integrity and suppressing neuroinflammation during the acute phase. Furthermore, our in vivo and in vitro analyses suggest that attenuation of ER stress reduces expression of chemokines by ECs, thereby alleviating neuroinflammatory responses and contributing to improved neurological outcomes.

## Materials and methods

### Animals

All animal experiments were approved by the Animal Care and Use Committee of Kanazawa University (approval number: AP234388). Mice were housed in a temperature- and humidity-controlled environment under a 12-h light/dark cycle with ad libitum access to food and water. All mice used in the study were male C57BL/6N, aged 10–12 weeks. To visualize IRE1 pathway activation in vivo, ERAI reporter mice (RBRC01099, RIKEN BioResource Center), which express a fluorescent XBP1-Venus fusion protein under ER stress conditions, were used [10, 14]. Ai14 (RCL-tdT)-D mice (JAX stock #007914, Jackson Laboratory) [15] and IRE1 floxed mice (*Ern1*^*flox/flox*^, RBRC05515, RIKEN BioResource Center) [12] were crossed with *Cdh*5*-BAC-Cre*^*ERT2*^ mice [16] to visualize Cre-targeted cells and to generate EC-specific IRE1 cKO mice (*Cdh*5*-BAC-Cre*^*ERT2*^;*Ern1*^*flox/flox*^, hereafter referred to as *Ern1*^*iΔEC*^), by tamoxifen-induced recombination. Tamoxifen (75 mg/kg, HY-13757A, MedChemExpress, Monmouth Junction, NJ) was administered intraperitoneally once daily for five consecutive days to both *Ern1*^*iΔEC*^ and control *Ern1*^*flox/flox*^ littermates, starting 12 days before surgery. In total, 32 wild-type (WT), 6 ERAI, 40 *Ern1*^*iΔEC*^, and 49 *Ern1*^*flox/flox*^ mice were used in this study.

### Traumatic brain injury (TBI)

TBI was induced using a cortical ablation method as previously described [17]. Mice were anesthetized via intraperitoneal injection of a cocktail containing Dorbene (0.75 mg/kg, Kyoritsu Seiyaku, Tokyo, Japan), Dormicam (4.0 mg/kg, Maruishi Pharmaceutical Co., Ltd., Osaka, Japan), and Vetorphale (5.0 mg/kg, Meiji Seika Pharma Co., Ltd., Tokyo, Japan). After confirming sufficient anesthesia, a midline scalp incision was made to expose the skull. Using bregma as a reference point, four stereotaxic points were marked: 2.0 mm anterior and 1.5 mm posterior to bregma, and 3.0 mm lateral to the left from each of these two points, forming a rectangular area. The skull within this area was carefully thinned and removed to expose the underlying cortex. The targeted cortical region, including the rostral and caudal forelimb areas of the motor cortex [17, 18], was aspirated to a depth of approximately 1.0 mm using a micropipette under microscopic visualization. Following aspiration, the skull bone was repositioned, and the scalp was sutured. Mice were placed on a heating pad during recovery from anesthesia and returned to their home cages once fully awake. Mice were randomly allocated to groups to ensure comparable initial body weight before TBI. Mice with excessive bleeding during the procedure were excluded. Outcome assessments were not blinded, but results were verified by independent investigators. Behavioral assessments were conducted on day –1 (baseline), 1, 3, 5, and 7 post-injury. A subset of mice was euthanized on day 1 for tissue collection used in immunohistochemistry, quantitative real-time PCR, and western blot analyses. Sham-operated mice, which underwent the same surgical procedures without cortical ablation, were used as behavioral controls. In all analyses except behavioral assessments, the contralateral (uninjured) cortex was used as an internal control.

### Tauroursodeoxycholic acid (TUDCA) treatment

To investigate the potential protective effects of TUDCA (HY-19696A; MedChemExpress) in TBI, WT and *Ern1*^*iΔEC*^ mice were randomly assigned to two groups: a vehicle control group (injected with sterile saline) and a TUDCA treatment group. TUDCA was administered intraperitoneally at a dose of 50 mg/kg once daily, starting immediately after surgery on the day of injury, and continuing throughout the experimental period.

### Cell culture

The mouse brain-derived endothelial cell line bEnd.3 (CRL-2299, ATCC, Manassas, VA) was cultured in Dulbecco’s Modified Eagle Medium (DMEM; Wako, Osaka, Japan) supplemented with 10% fetal bovine serum and 100 μg/mL penicillin-streptomycin. Cells were maintained at 37 °C in a humidified incubator with 5% CO_2_ and were not tested for mycoplasma during this study. At 24-h seeding, cells were serum-starved for 8 h before stimulation with lipopolysaccharide (LPS; 5 μg/mL) and TUDCA (0.1 mM or 0.2 mM) for 6 h.

### Immunohistochemistry

Mice were deeply anesthetized and perfused transcardially with cold phosphate-buffered saline, followed by 4% paraformaldehyde. Brains were carefully dissected and post-fixed in 4% paraformaldehyde at 4 °C overnight. For VE-cadherin staining, brains were post-fixed for 30 min at 4 °C to preserve membrane antigenicity. The brains were then cryoprotected in 30% sucrose in phosphate buffer at 4 °C for 2 days and were coronally sectioned at 30 μm thickness using a cryostat (Leica Biosystems, Wetzlar, Germany). Sections were blocked with 3% bovine serum albumin in 0.3% Triton X-100 for 1 h at room temperature and were incubated overnight at 4 °C with the following primary antibodies: anti-GFP (598, MBL, Tokyo, Japan; RRID:AB_591819, 1:1000), anti-CD31 (550274, BD Pharmingen, Franklin Lakes, NJ; RRID:AB_393571, 1:100), anti-VE-cadherin (AF1002, R&D Systems, Abingdon, UK; RRID:AB_2077789, 1:100), anti-CXCL10 (AF-466-NA, R&D Systems; RRID:AB_2292487, 1:200), anti-CD45 (550539, BD Pharmingen; RRID:AB_2174426, 1:200), anti-Laminin (L9393, Sigma, St. Louis, MO; RRID:AB_477163, 1:4000), anti-NeuN (24307, Cell Signaling Technology, Danvers, MA, USA; RRID:AB_2651140), and anti-Iba1 (ab107159, Abcam, Cambridge, UK; RRID:AB_10972670, 1:1000; and 019-19741, Wako; RRID:AB_839504, 1:500). anti-ERG (ab92513, Abcam, Cambridge, UK; RRID:AB_2630401, 1:800), anti-SMI31 (NE1022, Millipore; RRID:AB_2043448, 1:800), anti-IRE1α (NB100-2323, Novus; RRID: AB_10145203, 1:500), anti-p62 (018-22141, Wako; RRID:AB_10658438, 1:500), Secondary antibodies conjugated to Alexa Fluor 488 or Cy3 were used for fluorescence detection. Nuclei were counterstained with DAPI. Fluorescent images were acquired using a confocal microscope (Eclipse TE2000U, Nikon, Tokyo, Japan) and Nikon EZ-C1 software.

### TUNEL staining

To quantify apoptotic neurons in the perilesional cortex at 24 h after TBI, TUNEL staining was performed in combination with immunostaining for the neuronal marker NeuN. Apoptotic cells were detected using the ApopTag® Fluorescein In Situ Apoptosis Detection Kit (Sigma-Aldrich, St. Louis, MO) according to the manufacturer’s instructions. Following TUNEL labeling, brain sections were incubated with a mouse anti-NeuN antibody and counterstained with DAPI. TUNEL⁺/NeuN⁺ double-positive cells were counted as described in the following section.

### Quantification of cell number and fluorescence intensity

Quantification of cell numbers based on immunofluorescence staining was performed in the lateral cortex adjacent to the lesion site. For each mouse, 2 or 3 randomly selected brain sections were analyzed, and the average value across sections was used as the representative value for that mouse. CD45 (pan-leukocyte marker)-positive cells were counted within a region of interest (ROI) measuring approximately 1.61 mm^2^ (1270 × 1270 µm). CD45⁺ cells within Laminin⁺ areas, and GFP⁺, GFP⁺/ERG⁺ double-positive cells were counted within a 0.4 mm^2^ ROI (639 × 639 µm). NeuN⁺ and TUNEL⁺/NeuN⁺ double-positive cells were similarly counted within a 1.61 mm^2^ ROI. Fluorescence intensity analysis was also conducted in the perilesional cortex. The integrated fluorescence intensity of IgG was measured within an 11.62 mm^2^ ROI (3612 × 3612 µm). Integrated fluorescence intensities of VE-cadherin and CXCL10 were quantified in a perilesional zone extending 200 µm laterally from the lesion edge (0.13 mm^2^ ROI, 200 × 639 µm). The damaged area was defined as the region surrounded by Iba1⁺ microglia with marked reduction of NeuN⁺ neurons, and its area was measured accordingly. All image analyses were performed using ImageJ software (version 1.51; RRID: SCR_003070).

### Quantitative RT-PCR

Total RNA was extracted from approximately 8 mm³ of the lateral perilesional cortex using QIAzol Lysis Reagent (QIAGEN, Hilden, Germany) and FASTGene™ RNA Basic Kit (Nippon Genetics, Japan) according to the manufacturer’s instructions. Complementary DNA was synthesized from 1 µg of total RNA using the High-Capacity cDNA Reverse Transcription Kit (Applied Biosystems, Warrington, United Kingdom). Quantitative real-time PCR (qPCR) was performed using gene-specific primers and Thunderbird SYBR qPCR Mix (Toyobo, Osaka, Japan), and quantified with QuantStudio™ Real-Time PCR software v1.6.1 (Thermo Fisher Scientific, Waltham, MA). The following target genes were analyzed: spliced X-box binding protein 1 (*sXbp1*), Protein tyrosine phosphatase, receptor type C (*Ptprc*; CD45), Interleukin-1 beta (*Il1b*; IL-1β), Tumor necrosis factor *(Tnf*; TNFα), C–X–C motif chemokine ligand 10 (*Cxcl10*), Cadherin 5 (*Cdh5*; VE-cadherin), C–X–C motif chemokine ligand 1 (*Cxcl1*), C–C motif chemokine ligand 2 (*Ccl2*), and C–C motif chemokine ligand 5 (*Ccl5*). Expression levels were normalized to *Gapdh*, and relative expression was calculated using the comparative Ct (ΔΔCt) method. All primer sequences are listed in Supplementary Table 1.

### Western blotting

Protein samples were extracted from approximately 8 mm^3^ of the lateral perilesional cortex using RIPA buffer supplemented with protease and phosphatase inhibitors. The RIPA buffer contained 10 mM Tris (pH 7.6), 1 mM EDTA, 150 mM NaCl, 1% NP-40, 0.1% SDS, 0.2% sodium deoxycholate, 1 mM PMSF, 1 µg/mL aprotinin, 10 mM NaF, and 1 mM Na_3_VO_4_. After quantification, proteins were separated by SDS-PAGE and transferred to PVDF membranes. Membranes were blocked and incubated with HRP-conjugated anti-mouse IgG antibody (Invitrogen; RRID: AB_10960845, 1:5000) to detect endogenous mouse IgG. As the brain tissue was not perfused, IgG included both extravasated and circulating components. GAPDH (Wako; RRID: AB_2814991, 1:2000) was used as a loading control. Protein bands were visualized using an ECL detection system (GE Healthcare, Chicago, IL) and quantified using ImageJ software (version 1.51; RRID: SCR_003070). Full and uncropped western blots are provided in the Supplementary Material.

### Behavioral studies

#### Rotarod test

Motor coordination and balance were evaluated using a rotarod apparatus (LE8500, Harvard Apparatus/Panlab, Barcelona, Spain). Prior to testing, mice underwent a 3-day habituation phase during which they were trained three times per day, with 10-min intervals between sessions. During each session, mice were placed on a rotating rod set at a constant speed of 6 rpm for 5 min. The test sessions were conducted on day –1 (baseline) and on days 1, 3, 5, and 7 after TBI surgery. In the test trials, mice were placed on the rod accelerating from 4 to 40 rpm, and the latency to fall was recorded. Each mouse underwent three trials per session, with 5-min intervals between trials. The mean latency from the three trials was used as the performance score at each time point.

#### Grid-walk test

Forelimb motor function was assessed using a metal grid platform (200 mm × 240 mm) with grid spacing of 1.5 cm × 1.5 cm. Mice were acclimated to the apparatus by freely exploring the platform for 3 min before testing. Forelimb movements were recorded from below using a digital camera, and foot faults of the impaired right forelimb were analyzed. A foot fault was defined as a failure to bear weight properly on a grid bar, resulting in the limb slipping off or falling through the grid. The number of right forelimb foot faults was quantified and normalized to the first 100 steps taken during the trial. The grid surface was cleaned with 75% ethanol between trials to ensure consistency in testing conditions.

### RNA-sequencing

The preparation of a cDNA library and sequencing was conducted by Rhelixa, Inc (Tokyo, Japan). Total RNAs were extracted from the ipsilateral cortical tissue of *Ern1*^*flox/flox*^ and *Ern1*^*iΔEC*^ mice following TBI (*n* = 3 per group). cDNA libraries were prepared using the NEBNext Ultra II Directional RNA Library Prep Kit (New England Biolabs, Ipswich, MA). Each library was sequenced with the use of a NovaSeq X Plus system (Illumina, San Diego, CA). The quality of the raw sequencing data was checked with FastQC (version 0.12.1), and trimming of the adapter sequences was performed with Trimmomatic (version 0.38). The total amount of each mRNA was calculated with the use of a series of programs, including HISAT2 (version 2.1.0), featureCounts (version 1.6.3), and DESeq2 (version 1.24.0). RNA-seq reads were mapped against the mouse (mm10) genome. Differentially expressed genes (DEGs) were detected with the thresholds of adjusted *p*-value (*P*_adj_) < 0.05 and |log2 Fold Change (FC)| ≥ 1. Gene ontology analysis was performed using Metascape web tools for DEGs, and Heatmaps were created from Z-scores of the normalized counts using the pheatmap package (version 1.0.12) in R (version 4.4.1, R Foundation for Statistical Computing). The volcano plot was generated using the EnhancedVolcano package (version 1.26.0) in R, the cut-off level is *P*_adj_ < 0.05 and |log2FC| ≥ 1 for each gene. The raw reads are available in the DNA Data Bank of Japan (DDBJ) with DDBJ Sequence Read Archive (DRA) accession number PRJDB38021.

### Statistical analysis

All statistical analyses were conducted using GraphPad Prism software (version 10.1.2). Quantitative data are presented as mean ± standard error of the mean (SEM). The number of samples (*n*), representing independent biological replicates, is indicated in the corresponding figure legends. Sample sizes were not predetermined statistically but were determined based on general standards in the field, and formal testing for normality was not performed. For comparisons between two groups, the Mann–Whitney *U*-test was applied. For comparisons among three or more groups, one-way analysis of variance (ANOVA) followed by Tukey’s multiple comparisons test, or two-way ANOVA followed by Sidak’s multiple comparisons test was used. All tests were two-sided. A *p*-value less than 0.05 was considered statistically significant.

## Results

### Endothelial IRE1 activation post-TBI and its contribution to neurological impairment

**Fig. 1:**
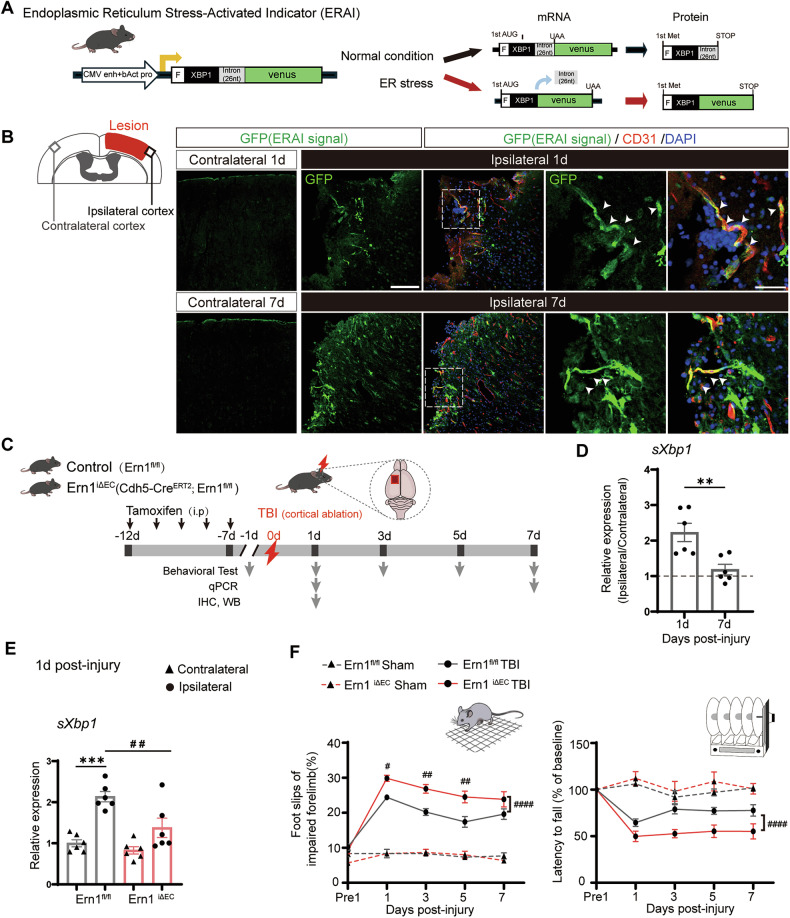
IRE1 signaling is activated in endothelial cells after TBI and contributes to functional outcomes. **A** Schematic diagram of ERAI transgenic mice, which express a Venus–XBP1s fusion protein upon IRE1-mediated Xbp1 mRNA splicing. **B** Representative immunofluorescence images of GFP (Venus–XBP1s, green) and CD31 (red) in the cortex of ERAI mice at day 1 and day 7 post-TBI. The red area in the schematic indicates the lesion, and the black and gray box corresponds to the magnified images in the ipsilateral and contralateral cortex, respectively. Arrowheads indicate GFP⁺CD31⁺ double-positive cells in the perilesional cortex. Scale bars: overview, 150 μm; inset, 50 μm. **C** Experimental design and generation of endothelial cell-specific IRE1 knockout (*Ern1*^*iΔEC*^) and control (*Ern1*^*flox/flox*^) mice. **D** qPCR analysis of spliced *Xbp1* (*sXbp1*) expression in the cortex at day 1 and day 7 post-TBI. **p < 0.01 by Mann–Whitney *U*-test (*n* = 6 per group). **E** Relative expression of *sXbp1* in ipsilateral and contralateral cortices. ***p < 0.001 vs. contralateral cortex; ##*p* < 0.01 vs. *Ern1*^*flox/flox*^ by one-way ANOVA with Tukey’s multiple comparisons test (*n* = 6). **F** Grid-walk and Rotarod performance over 7 days after TBI. Two-way ANOVA within the TBI groups (*n* = 6 per genotype), with Sidak’s post hoc test. Grid-walk test (genotype: *p* < 0.0001, time: *p* < 0.0001, genotype × time: *p* < 0.05, post hoc test following interaction, day 1: *p* < 0.05, day 3, 5: *p* < 0.01). Rotarod test (genotype: *p* < 0.0001, time: *p* < 0.0001, genotype × time: *p* > 0.05). Sham groups (*n* = 3 per genotype) are displayed as reference. Data are presented as mean ± SEM.

To visualize IRE1 pathway activation in vivo, we utilized ERAI transgenic mice that express a Venus-fused XBP1s protein upon IRE1-mediated *Xbp1* splicing (Fig. 1A) [10, 14]. Previously, we reported early ERAI signal induction in ECs following stab injury and cerebral ischemia [11]. Consistent with these findings, we observed strong GFP-positive signals predominantly in the perilesional cortex on Day 1 post-injury. These signals colocalized with the EC marker CD31, and this colocalization persisted, at least partially, on Day 7 (Fig. 1B). To quantitatively assess endothelial IRE1 pathway activation over time, we performed immunohistochemistry using an anti-ERG antibody to label EC nuclei. Quantification revealed that the percentage of ERG⁺GFP⁺ cells among GFP⁺ cells was significantly higher on Day 1 than on Day 7 post-injury (Supplementary Fig. S1A).

To elucidate the functional role of endothelial IRE1 signaling in this context, we generated tamoxifen-inducible, EC-specific IRE1 cKO mice (*Ern1*^*iΔEC*^, *Cdh5*^CreERT2^; *Ern1*^flox/flox^) and subjected them to the same cortical ablation TBI procedure (Fig. 1C). Endothelial-specific deletion of IRE1 was confirmed by reporter expression in CD31⁺ cells using *Cdh5*^CreERT2^; Ai14 (RCL-tdT)-D mice and by reduced IRE1 immunostaining in ECs of *Ern1*^*iΔEC*^ mice (Supplementary Fig. S1B–D). Quantitative PCR revealed marked induction of *sXbp1* expression in the ipsilateral cortex on Day 1 post-injury, which declined by Day 7 (Fig. 1D). Notably, this upregulation was significantly less prominent in *Ern1*^*iΔEC*^ mice than in their *Ern1*^*flox/flox*^ littermates (Fig. 1E). We found no significant differences between genotypes in terms of expression of *sXbp1* in the contralateral cortex. Behavioral assessments revealed significant motor impairment after TBI. *Ern1*^*iΔEC*^ mice exhibited more severe deficits than controls in both the grid-walk and rotarod tests (Fig. 1F), with no differences observed under sham conditions. These findings indicate that endothelial IRE1 is activated following TBI and mitigates post-injury motor dysfunction.

### Deficiency of endothelial IRE1 exacerbates vascular permeability after TBI

**Fig. 2:**
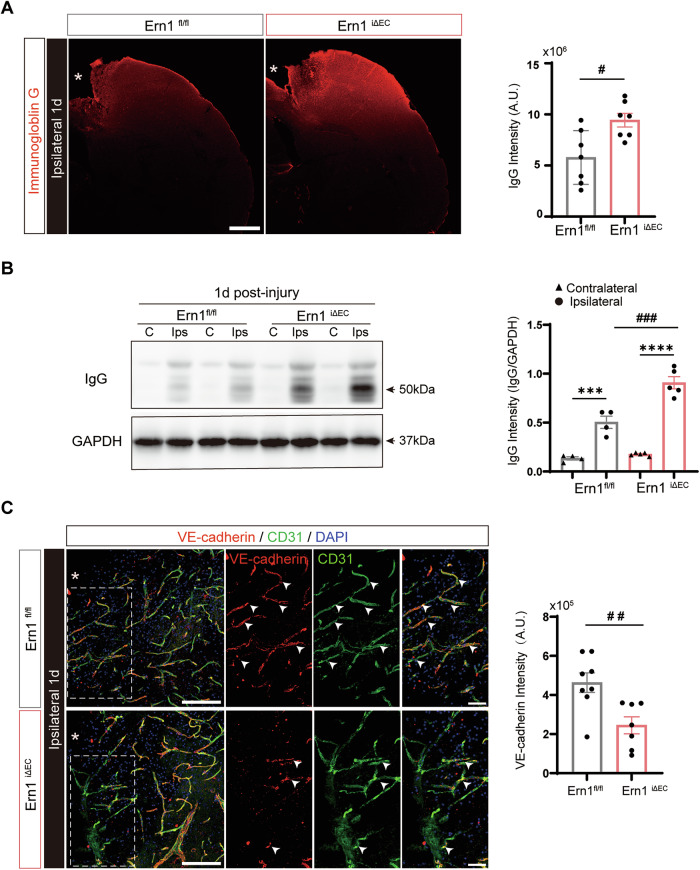
Endothelial IRE1 deficiency disrupts BBB integrity and increases vascular permeability after TBI. **A** Representative images showing IgG (red) extravasation in the ipsilateral cortex of *Ern1*^*flox/flox*^ and *Ern1*^*iΔEC*^ mice at day 1 post-TBI. Asterisks denote the lesion site. Scale bar: 500 μm. Right, quantification of IgG fluorescence intensity. #*p* < 0.05 by Mann–Whitney *U*-test (*n* = 7). **B** Western blot analysis and quantification of IgG in ipsilateral and contralateral cortices. ***p < 0.001, ****p < 0.0001 vs. contralateral; ###*p* < 0.001 vs. *Ern1* ^*flox/flox*^ by one-way ANOVA with Tukey’s test (*Ern1*^*flox/flox*^, *n* = 4; *Ern1*^*iΔEC*^, *n* = 5). **C** Representative immunofluorescence images of CD31 (green) and VE-cadherin (red) in the perilesional cortex. The boxed area is shown at higher magnification. Arrowheads indicate CD31⁺VE-cadherin⁺ cells; asterisks indicate the lesion site. Scale bars: overview, 150 μm; inset, 50 μm. Right, quantification of VE-cadherin fluorescence within a 200 μm perilesional region. ##*p* < 0.01 by Mann–Whitney *U*-test (*Ern1*^*flox/flox*^, *n* = 8; *Ern1*^*iΔEC*^, *n* = 7). Data are presented as mean ± SEM.

Building on our findings of early IRE1 activation in ECs after TBI and the well-established evidence that TBI rapidly disrupts the BBB via endothelial injury [19, 20], we next investigated whether endothelial IRE1 contributes to BBB integrity. To do this, we assessed vascular permeability by evaluating extravasation of endogenous IgG, a plasma protein that normally does not cross the BBB. Immunohistochemical analysis on Day 1 post-injury revealed widespread extravasation of IgG in the ipsilateral cortex, centered around the lesion site. IgG immunoreactivity in *Ern1*^*iΔEC*^ mice was significantly higher than that in *Ern1*^*flox/flox*^ mice (Fig. 2A). Consistent with this, western blot analysis demonstrated increased IgG levels in the ipsilateral cortex of *Ern1*^*iΔEC*^ mice relative to those of *Ern1*^*flox/flox*^ mice, although no genotype-dependent differences were observed in the contralateral cortex (Fig. 2B).

To investigate the mechanisms by which endothelial IRE1 deficiency increases vascular permeability, we performed immunohistochemistry to detect CD31, a pan-endothelial marker, and VE-cadherin, a critical adherens junction protein essential for endothelial barrier integrity. Expression of VE-cadherin in the perilesional cortex on Day 1 post-injury was markedly lower in *Ern1*^*iΔEC*^ mice than in *Ern1*^*flox/flox*^ mice, suggesting compromised endothelial junction integrity (Fig. 2C). By contrast, qualitative assessment of CD31 expression revealed no overt differences between genotypes, suggesting that overall vascular density was largely preserved. These findings suggest that loss of endothelial IRE1 impairs barrier integrity by downregulating VE-cadherin, leading to increased vascular leakage following TBI.

### Endothelial IRE1 deficiency promotes immune cell infiltration and neuroinflammation after TBI

**Fig. 3:**
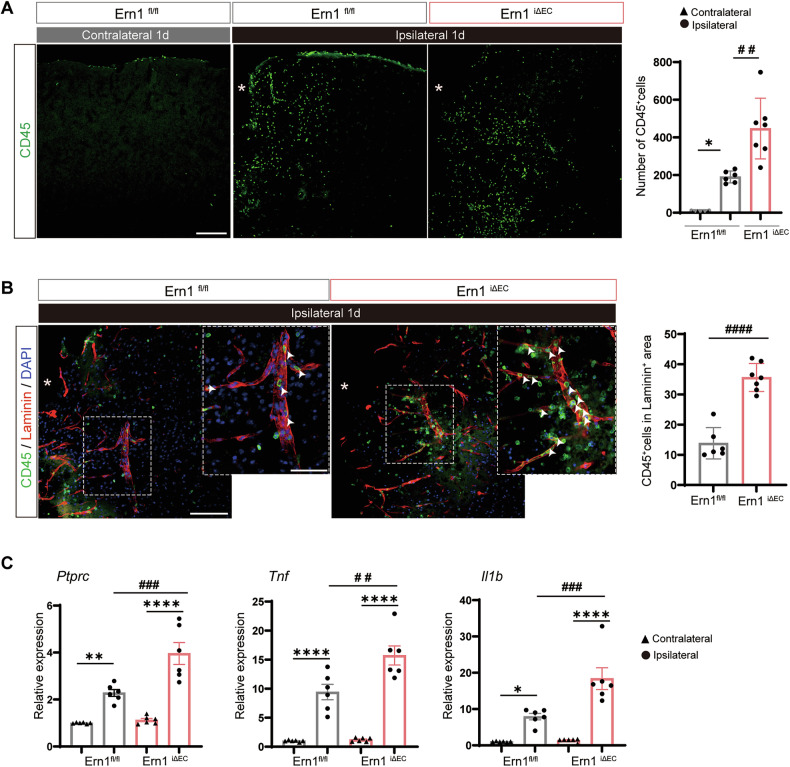
Endothelial IRE1 deficiency increases immune cell infiltration and inflammatory response after TBI. **A** Representative images of CD45⁺ immune cells (green) in the cortex of *Ern1*^*flox/flox*^ and *Ern1*^*iΔEC*^ mice at day 1 post-TBI. Asterisks indicate the lesion site. Scale bar: 200 μm. Right, quantification of CD45⁺ cells. *p < 0.05 vs. contralateral; ##*p* < 0.01 vs. *Ern1*^*flox/flox*^ by one-way ANOVA with Tukey’s test (*Ern1*^*flox/flox*^, *n* = 6; *Ern1*^*iΔEC*^, *n* = 7). **B** Double immunofluorescence staining for CD45 (green) and laminin (red). The boxed region is shown at higher magnification. Arrowheads indicate CD45⁺ cells associated with vessels. Scale bars: overview, 200 μm; inset, 100 μm. Right, quantification of CD45⁺Laminin⁺ cells. ####*p* < 0.0001 by Mann–Whitney *U*-test (*Ern1*^*flox/flox*^, *n* = 6; *Ern1*^*iΔEC*^, *n* = 7). **C** RT-qPCR analysis of *Ptprc* (CD45), *Tnf*, and *Il1b* expression in ipsilateral and contralateral cortices at day 1 post-TBI. *p < 0.05, **p < 0.01, ****p < 0.001 vs. contralateral; ##*p* < 0.01, ###*p* < 0.001 vs. *Ern1* ^*flox/flox*^ by one-way ANOVA with Tukey’s test (*n* = 6). Data are presented as mean ± SEM.

Given that endothelial deficiency of IRE1 exacerbated BBB disruption after TBI, we next assessed the extent of immune cell infiltration and associated inflammatory responses in the perilesional cortex. On Day 1 post-injury, we noted extensive infiltration of the ipsilateral cortex parenchyma by CD45⁺ cells, which was in contrast to their confinement to surface and perivascular regions within the contralateral cortex (Fig. 3A). *Ern1*^*iΔEC*^ mice showed a broader distribution, as well as a significantly greater number of CD45⁺ cells in the perilesional cortex than *Ern1*^*flox/flox*^ mice. To evaluate the interaction between infiltrating immune cells and the vasculature, we performed double immunostaining to detect CD45 and the vascular basement membrane marker laminin. In *Ern1*^*iΔEC*^ mice, a larger proportion of CD45⁺ cells were located adjacent to laminin⁺ vessels. Quantification confirmed a significant increase in the number of CD45⁺ cells within laminin⁺ regions in *Ern1*^*iΔEC*^ mice relative to *Ern1*^*flox/flox*^ mice (Fig. 3B). Next, we evaluated expression of inflammatory genes by RT-qPCR. Expression of *Ptprc* (CD45), *Tnf* (TNF-α), and *Il1b* (IL-1β) was upregulated in the ipsilateral cortex after TBI, and significantly higher levels were observed in *Ern1*^*iΔEC*^ mice than in controls (Fig. 3C). No genotype-dependent differences were detected in the contralateral cortex. These results indicate that endothelial IRE1 restrains immune cell infiltration, as well as production of inflammatory cytokines, following TBI.

### Deficiency of endothelial IRE1 expands the area of neurodegeneration after TBI

**Fig. 4:**
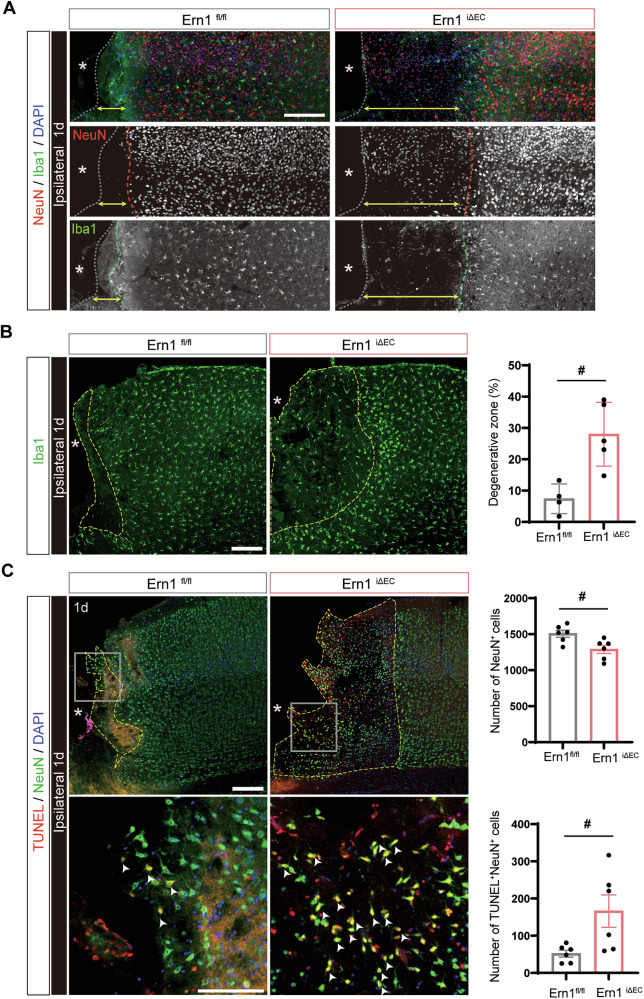
Endothelial IRE1 deficiency enlarges the neurodegenerative area and increases neuronal apoptosis. **A** Representative images of NeuN (red) and Iba1 (green) immunostaining in the ipsilateral cortex of *Ern1*^*flox/flox*^ and *Ern1*^*iΔEC*^ mice at day 1 post-TBI. Red and green lines indicate the borders of NeuN signal reduction and Iba1⁺ microglial clustering, respectively. Asterisks and gray lines indicate the lesion site and its edge. Yellow double-headed arrows demarcate the perilesional degenerative zone, which lies between these boundaries. Scale bar: 250 μm. **B** Iba1 immunostaining in the ipsilateral cortex on day 1 post-TBI. Yellow dashed lines delineate the perilesional degenerative zone surrounded by clustered Iba1⁺ cells. Scale bar: 200 μm. Right, quantification of the damaged area. #*p* < 0.05 by Mann–Whitney *U*-test (*Ern1*^*flox/flox*^, *n* = 4; *Ern1*^*iΔEC*^, *n* = 5). **C** TUNEL (red) and NeuN (green) double staining in the ipsilateral cortex. Arrowheads indicate TUNEL⁺NeuN⁺ apoptotic neurons. Asterisks indicate the lesion site; yellow lines denote the border of the perilesional degenerative zone. Scale bars: overview, 200 μm; inset, 100 μm. Right, quantification of NeuN⁺ cells, TUNEL⁺NeuN⁺ cells per field in overview images. #*p* < 0.05 by Mann–Whitney *U*-test (*n* = 6). Data are presented as mean ± SEM.

Neuroinflammation triggered by TBI is a known contributor to neuronal apoptosis [21, 22]. Given that deficiency of endothelial IRE1 exacerbates inflammatory responses, we next evaluated its impact on neuronal damage. First, we performed immunostaining to detect NeuN, a neuronal marker, and Iba1, a microglial marker, on Day 1 post-injury. At the inner edge of the perilesional cortex, the number of NeuN⁺ neurons was reduced, and the remaining neurons showed decreased NeuN expression with somatic atrophy, as demonstrated by NeuN and the neurofilament marker SMI31 (Supplementary Fig. S2A). By contrast, there was a dense accumulation of activated Iba1⁺ microglia around this area. The region delineated by these boundaries and the lesion edge, defined as the perilesional degenerative zone, is indicated by double-headed arrows in Fig. 4A. This zone appeared visibly expanded in *Ern1*^*iΔEC*^ mice compared with *Ern1*^*flox/flox*^ mice. To quantify the extent of neurodegeneration, we used a high-sensitivity Iba1 antibody to delineate microglial boundaries and measured the perilesional degenerative zone outlined by the yellow dotted lines. Quantitative analysis revealed a significantly larger area of damage in *Ern1*^*iΔEC*^ mice than in controls (Fig. 4B). We then assessed neuronal apoptosis using TUNEL staining combined with NeuN immunofluorescence. Numerous TUNEL⁺ cells were detected in the perilesional degenerative zone, and most colocalized with NeuN signals, indicating apoptotic neurons (Fig. 4C). The number of NeuN⁺ cells was significantly reduced, and TUNEL⁺/NeuN⁺ double-positive cells were significantly increased in *Ern1*^*iΔEC*^ mice compared with controls. In addition to apoptosis, we evaluated autophagy using the marker p62. Immunohistochemistry for p62 with CD31 co-labeling revealed no significant differences in signal intensity for total p62, vascular (CD31⁺) p62, or non-vascular (CD31^−^) p62 between *Ern1*^*iΔEC*^ and *Ern1*^*flox/flox*^ controls (Supplementary Fig. S2B). Taken together, these results suggest that endothelial IRE1 limits the expansion of neurodegeneration and neuronal apoptosis after TBI.

### Deficiency of endothelial IRE1 upregulates expression of CXCL10 following TBI

**Fig. 5:**
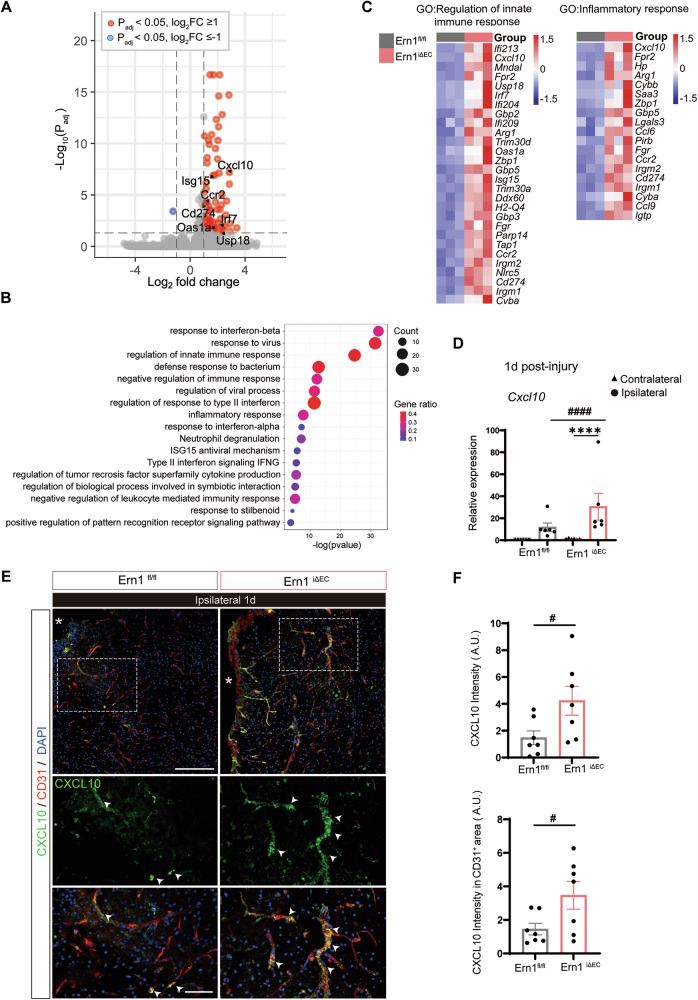
Endothelial IRE1 deficiency enhances CXCL10 expression after TBI. **A** Total RNA isolated from ipsilateral cortices from *Ern1*^*iΔEC*^ mice and *Ern1*^*flox/flox*^ control mice on day 1 post-injury was subjected to RNA-sequencing (*n* = 3 per group). Volcano plot of DEGs in *Ern1*^*iΔEC*^ group compared with the *Ern1*^*flox/flox*^ group (*P*_adj_ < 0.05, |log2FC| ≥ 1). **B** Significant enrichment of GO terms in *Ern1*^*iΔEC*^ mice by GO enrichment analysis of 73 DEGs. **C** Heatmaps show gene expressions in the GO terms “Regulation of innate immune response” and “Inflammatory response”. Color scale bars indicate Z score by row. **D** RT-qPCR analysis of *Cxcl10* expression in cortices of *Ern1*^*flox/flox*^ and *Ern1*^*iΔEC*^ mice at day 1 post-TBI. ****p < 0.0001 vs. contralateral; ####*p* < 0.0001 vs. *Ern1* ^*flox/flox*^ by one-way ANOVA with Tukey’s test (*n* = 6). **E** Immunofluorescence staining of CXCL10 (green) and CD31 (red) in the ipsilateral cortex. The boxed area is magnified below. Arrowheads indicate CXCL10⁺CD31⁺ double-positive cells; Asterisks indicate the lesion site. Scale bars: overview, 150 μm; inset, 50 μm. **F** Quantification of CXCL10 intensity and the intensity in the CD31⁺ vascular area within a 200 μm perilesional region. #*p* < 0.05 by Mann–Whitney *U*-test (*n* = 7). Data are presented as mean ± SEM.

To gain insight into endothelial IRE1 contributions to TBI pathogenesis, we performed bulk RNA-sequencing on the ipsilateral cortices from *Ern1*^*iΔEC*^ and *Ern1*^*flox/flox*^ controls at 1d-post-injury. There were 73 DEGs (*P*_adj_ < 0.05, |log2FC | ≥1), with 72 upregulated genes and 1 downregulated gene (Fig. 5A, Supplementary Table S2). GO enrichment analysis revealed the DEG set was significantly enriched for interferon/innate immune pathways, including “response to interferon-beta”, “response to virus”, “regulation of innate immune response”, and “inflammatory response” (Fig. 5B). We observed upregulation of the chemokine axis (*Cxcl10*, *Ccr2*), interferon transcriptional control (*Irf7*, *Usp18*), canonical interferon-stimulated genes (*Isg15*, *Oas1a*), and the checkpoint pathway (*Cd274*/PD-L1). Heatmap of DEGs from two GO terms, including the above genes were shown in Fig. 5C. Several DEGs (e.g., *Cxcl10*, *Isg15*, *Irf7*, *Usp18*, *Bst2*) are known to be inducible in ECs by interferons [23–25], whereas others (e.g., *Ccr2*, *Arg1*, *Lgals3*, *Cybb*) likely reflect increased leukocyte content in bulk tissue. Notably, *Cxcl10* was highly ranked (top-18 by *P*_adj_; top-5 by fold change) among the upregulated 72 DEGs and included in 7 of 17 enriched pathways (Fig. 5B, Supplementary Table S2). Since CXCL10 contributes to the inflammatory cascade by promoting leukocyte adhesion and increasing BBB permeability [23, 26], we focused on CXCL10 as a plausible effector associated with the exacerbated immune recruitment and barrier dysfunction in *Ern1*^*iΔEC*^ mice. qPCR validated significantly upregulated expression of *Cxcl10* in the cortex on Day 1 post-injury of *Ern1*^*iΔEC*^ mice than in *Ern1*^*flox/flox*^ mice (Fig. 5D). Given the established role of other chemokines after TBI [27, 28], we assessed expression levels of *Cxcl1*, *Ccl2*, and *Ccl5*, showing that *Ccl2* expression was significantly higher in *Ern1*^*iΔEC*^ mice than in *Ern1*^*flox/flox*^ mice, whereas there *Cxcl1* and *Ccl5* showed no significant differences (Supplementary Fig. S3A). Next, we performed immunohistochemistry to determine the cellular source of CXCL10. Strong expression of CXCL10 was induced in the perilesional cortex at Day 1 post-TBI, and showed prominent colocalization with the endothelial marker CD31 (Fig. 5E). Quantitative analysis of both total CXCL10⁺ signal intensity and CXCL10⁺ signal intensity in CD31-positive vascular area confirmed a significant increase in expression in *Ern1*^*iΔEC*^ mice relative to that in controls (Fig. 5F). These findings suggest that endothelial IRE1 negatively regulates expression of CXCL10 after TBI.

### A chemical chaperone suppresses endothelial expression of CXCL10 and improves motor outcomes after TBI

**Fig. 6:**
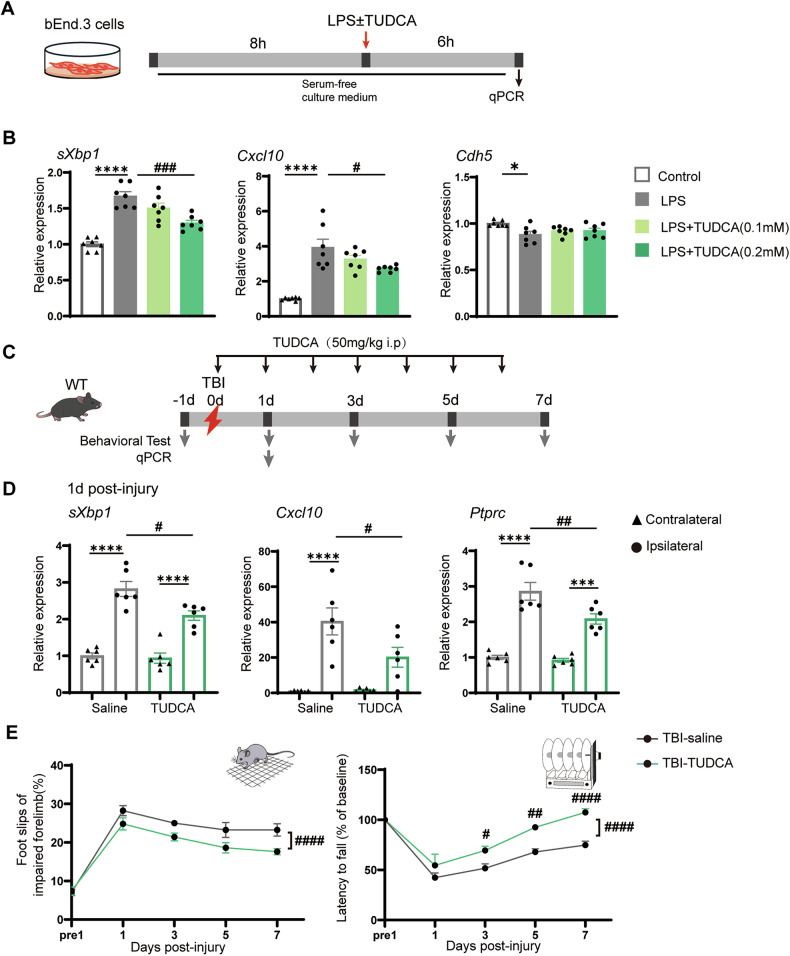
TUDCA attenuates endothelial chemokine expression and improves motor outcomes after TBI. **A** Schematic of the bEnd.3 cell culture experiment. **B** RT-qPCR analysis of *sXbp1*, *Cxcl10*, and *Cdh5* (VE-cadherin) in *bEnd.3* cells treated as indicated. *p < 0.05, ****p < 0.0001 vs. control; #*p* < 0.05, ###*p* < 0.001 vs. LPS by one-way ANOVA with Tukey’s test (*n* = 7). **C** Experimental timeline of TUDCA administration after TBI. **D** RT-qPCR analysis of *sXbp1*, *Cxcl10*, and *Ptprc* (CD45) in cortices of mice with TUDCA or saline administration at day 1 post-TBI. ***p < 0.001, ****p < 0.0001 vs. contralateral; #*p* < 0.05, ##*p* < 0.01 vs. TBI-saline by one-way ANOVA with Tukey’s test (*n* = 6). **E** Grid-walk and rotarod performance over 7 days after TBI. Two-way ANOVA with Sidak’s post hoc test (TBI-saline: *n* = 5; TBI-TUDCA: *n* = 4). Grid-walk test (group: *p* < 0.0001, time: *p* < 0.001, group × time: *p* > 0.05). Rotarod test (group: *p* < 0.0001, time: *p* < 0.0001, group × time: *p* < 0.05, post hoc test following interaction, day 3: *p* < 0.05, day 5: *p* < 0.01, day 7: *p* < 0.0001). Data are presented as mean ± SEM.

Given the increased expression of CXCL10 observed in EC-specific IRE1 cKO mice, we hypothesized that ER stress regulates expression of CXCL10 in brain ECs. To test this, we treated bEnd.3 cells with LPS to mimic inflammatory stress and then assessed the impact of the chemical chaperone TUDCA, which alleviates ER stress, on expression of *Cxcl10* (Fig. 6A, Supplementary Fig. S4A). qPCR analysis showed that LPS significantly induced expression of *sXbp1* and *Cxcl10*. In LPS-treated cells, TUDCA attenuated the induction of both *sXbp1* and *Cxcl10* in a dose-dependent manner, with 0.2 mM producing a significant reduction (Fig. 6B). LPS also decreased expression of *Cdh5* (VE-cadherin), although this effect was not clearly reversed by TUDCA. Treatment with the ER stress inducers tunicamycin or thapsigargin also increased *Cxcl10* expression (Supplementary Fig. S4B). Together, these data indicate that the level of ER stress in ECs regulates *Cxcl10* expression in vitro, with relief of ER stress attenuating its induction.

To evaluate the in vivo relevance of these findings, we administered TUDCA to WT mice after TBI (Fig. 6C). On Day 1 post-injury, expression of *sXbp1*, *Cxcl10*, and *Ptprc* (CD45) in the ipsilateral cortex was significantly lower in the TUDCA-treated group than in controls (Fig. 6D). Immunohistochemical analysis revealed that TUDCA administration significantly reduced the number of Iba1⁺ cells in the perilesional cortex, consistent with the above findings, while having no significant effect on the number of NeuN-positive cells (Supplementary Fig. S4C, D). Behavioral assessments revealed that TUDCA-treated mice showed improved motor performance in both the rotarod and grid-walk tests (Fig. 6E). TUDCA treatment in *Ern1*^*iΔEC*^ mice also had effects on motor performance; however, it had no significant effect on the expression of the gene mentioned above, indicating only partial improvement under this condition (Supplementary Fig. S3 F, G).

Taken together, these results suggest that chemical attenuation of ER stress suppresses the induction of CXCL10 and improves neurological dysfunction after TBI.

## Discussion

**Fig. 7:**
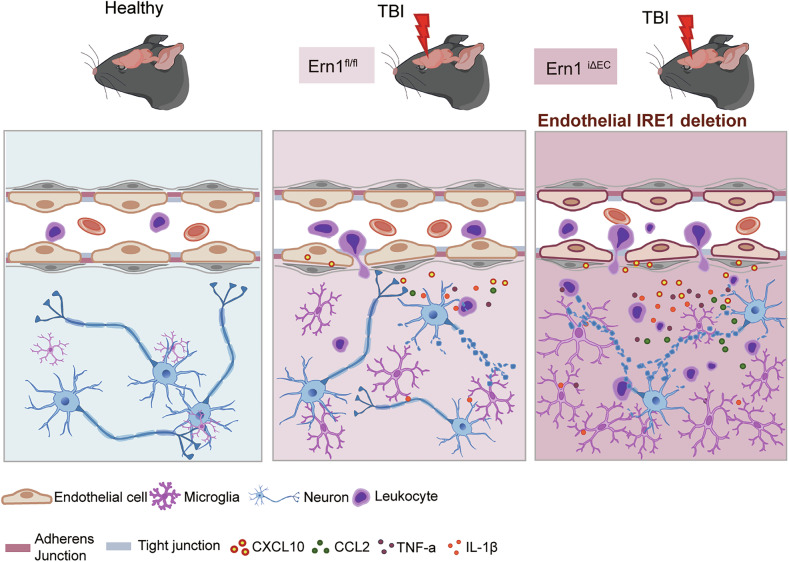
Proposed model: Endothelial IRE1 signaling preserves BBB function and suppresses inflammation after TBI. Loss of IRE1 in brain endothelial cells impairs VE-cadherin-mediated junctions and increases BBB permeability, facilitating immune cells infiltration into the brain parenchyma. This is accompanied by endothelial chemokines expression, including CXCL10, which promotes further immune cell recruitment. The accumulation of immune cells amplifies inflammation and contributes to neuronal apoptosis.

IRE1 signaling has been studied extensively in neurons and glial cells, providing a molecular basis for therapeutic strategies in neurological diseases [7–9, 29, 30]. However, its function in ECs within the context of neurological disorders remains poorly characterized. In this study, we investigated the EC-specific role of IRE1 signaling in a TBI model using conditional genetic manipulation targeting vascular ECs. Our findings demonstrate that EC-specific deletion of IRE1 exacerbates vascular leakage, immune cell infiltration, neurodegeneration, and neurological deficits. Furthermore, we identified CXCL10 as an endothelial chemokine linked to the phenotype in endothelial IRE1 deficiency and found that modulation of endothelial ER stress regulates expression of CXCL10. These results reveal a previously underappreciated but critical role of endothelial IRE1 signaling in limiting neurovascular injury and inflammation following TBI (Fig. 7).

We observed increased extravasation of IgG and reduced expression of VE-cadherin in *Ern1*^*iΔEC*^ mice, indicating that IRE1 is required for maintaining BBB integrity after TBI (Fig. 2A–C). Importantly, we found no significant changes in the contralateral cortex, suggesting that endothelial IRE1 becomes critical under pathological conditions rather than physiological conditions. Previous studies implicated the IRE1 pathway in endothelial angiogenic responses by showing that IRE1 knockdown impairs VEGF-induced tube formation and choroidal neovascularization [31], and that EC-specific deletion of XBP1 disrupts vascular growth in the retina and hinders perfusion recovery after hindlimb ischemia, effects that are reversed by reconstitution of XBP1s [13]. XBP1s promote survival, migration, and angiogenesis of brain microvascular ECs under ischemic stress [32]. While these studies focused primarily on angiogenesis, our results demonstrate a distinct, angiogenesis-independent function of IRE1 in preserving endothelial junctional integrity during the acute phase of TBI. This aligns with reports showing that chemical chaperones preserve tight junctions after spinal cord injury [33] and that pharmacological inhibition of XBP1 decreases expression of tight junction proteins by epithelial cells [34]. Our in vivo genetic approach uniquely demonstrates that IRE1 signaling plays a protective role in BBB stability post-TBI, highlighting its therapeutic potential for acute CNS injuries.

Deletion of IRE1 from ECs led to increased infiltration by CD45⁺ cells, increased expression of inflammatory cytokines, and expansion of the perilesional degenerative zone surrounded by microglia (Figs. 3 and 4), suggesting amplified neuroinflammation. Although immune responses help to clear debris post-TBI, excessive or prolonged activation aggravates neuronal damage and impairs recovery [35]. In our model, the intensified immune response likely contributed to the worse neurological outcomes of *Ern1*^*iΔEC*^ mice (Fig. 1F). The IRE1–XBP1 pathway in immune cells is usually associated with pro-inflammatory responses: indeed, deletion of IRE1 from myeloid cells attenuates inflammatory arthritis in mice [36], ablation of XBP1 in macrophages reduces TLR-induced cytokines [37], and knockdown of XBP1 in astrocytes mitigates inflammation in a multiple sclerosis model [9]. Taken together with our findings, the findings of these previous studies raise the possibility that IRE1 signaling may exert cell type-specific effects, e.g., by promoting inflammation in immune cells, but limiting it in ECs by restricting immune cell trafficking. Comparative studies employing cell type-specific knockouts within the same injury models may clarify these contrasting roles.

Our transcriptomic analysis supports this interpretation. Bulk RNA-sequencing of ipsilateral cortices from *Ern1*^*iΔEC*^ mice showed that most DEGs belonged to interferon/innate immune pathways and included several genes known to be interferon-inducible in ECs, such as *Cxcl10*, *Isg15*, *Irf7*, *Usp18*, and *Bst2* (Fig. 5A–C) [23–25]. Within the DEG set, *Cxcl10* ranked highly and is positioned to link endothelial interferon activation to enhanced leukocyte recruitment. Because bulk RNA-sequencing can be influenced by increased leukocyte infiltration, we cannot exclude that other endothelial effectors were masked by the dominantly upregulated inflammatory transcripts. Nevertheless, the localization of CXCL10 in CD31^+^ ECs in immunohistochemistry (Fig. 5E) supports an endothelial contribution. Future work using single-cell RNA-sequencing will be required to understand the signaling more precisely.

A recent study shows that EC-derived CXCL10 facilitates T cell adhesion in cerebral malaria [23], highlighting its pathological relevance. The same study also shows that CXCL10 was predominantly upregulated in brain ECs in *Cxcl10*-BFP reporter mice, further supporting our findings. In our study, deletion of endothelial IRE1 increased expression of *Cxcl10* in the injured cortex significantly (Fig. 5D), suggesting that IRE1 negatively regulates the chemokines in ECs. Our findings that the chemical chaperone TUDCA suppressed expression of *Cxcl10* both in vitro and in vivo (Fig. 6B, D) and that ER stress inducers increased *Cxcl1*0 expression in vitro (Supplementary Fig. S4B) are consistent with prior findings that TUDCA attenuates ER stress- or ischemia-induced expression of CXCL10 in retinal cells [38, 39]. Interestingly, PERK knockdown reduces production of CXCL10 and CCL2 by inhibiting the NF-κB and STAT3 pathways in photoreceptor cells, while knockdown of XBP1 has the opposite effect [39]. These findings support our hypothesis that IRE1 attenuates chemokine expression in ECs. Moreover, TUDCA also improved motor deficits significantly following TBI (Fig. 6E), a finding consistent with its neuroprotective effects in models of spinal cord injury [33, 40], Parkinson’s disease [41], and Alzheimer’s disease [42]. Previous studies show that TUDCA suppresses microglial proliferation, migration, and morphological activation [43, 44], potentially by inhibiting NF-κB signaling, which is activated under ER stress conditions [43, 45, 46]. In our study, TUDCA reduced expression of CXCL10 by both cultured ECs and the injured cortex, accompanied by decreased immune activation and improved behavioral outcomes (Fig. 6B, D, and E); however, TUDCA provided a partial benefit on the phenotypes such as CXCL10 expression in *Ern1*^*iΔEC*^ mice (Supplementary Fig. S4E–G). Taken together with the previous report that ER stress-induced CXCL10 is promoted by PERK, whereas restrained by IRE1 [39], our findings suggest that loss of IRE1 in ECs may shift the balance toward PERK-dependent CXCL10 induction, potentially resulting in a partial therapeutic effect with TUDCA. Given that CXCL10 is a downstream target of NF-κB [47], our findings suggest that TUDCA alleviates ER stress-induced inflammation, at least in part, by inhibiting NF-κB activation and downregulating expression of CXCL10 in brain ECs. However, TUDCA likely affects multiple CNS cell types, so further studies will be needed to clarify its cell type-specific mechanisms of action.

This study has several limitations. First, the molecular mechanism by which endothelial IRE1 regulates the expression of CXCL10 remains unclear. Second, although we targeted ECs genetically, systemic deletion cannot fully exclude contributions from other vascular beds. Third, our cortical ablation model, which offers advantages in reproducibility and precise lesion control, does not replicate the biomechanical stresses of impact-based TBI models such as controlled cortical impact [48]. Future studies using clinically relevant impact-based TBI models would be important to validate our findings across different injury types.

In conclusion, this study provides the first in vivo evidence that endothelial IRE1 signaling protects the brain after TBI by preserving BBB integrity and suppressing inflammation. Given the therapeutic accessibility of brain ECs, targeting ER stress pathways in the vasculature may represent a promising strategy for mitigating secondary injury after TBI.

## Supplementary information

Supplementary Information

Original WB data

Supplementary Table S2

## Data Availability

All data needed to evaluate the conclusions in the paper are present in the paper and/or the Supplementary Materials.
