# Supplementary material for: Endothelial IRE1 signaling maintains blood–brain barrier integrity and limits neuroinflammation after traumatic brain injury
Source: Cell Death Dis. 2026 Feb 9;17(1):210. doi: 10.1038/s41419-026-08461-2 (PMC12921219; doi:10.1038/s41419-026-08461-2)
Supplement: Supplementary file 1 — Supplementary Information [file 41419_2026_8461_MOESM1_ESM.pdf]

Supplementary information

Title: Endothelial IRE1 signaling maintains blood–brain barrier integrity and limits neuroinflammation after traumatic brain injury

Authors: Qiyan Fan[1], Mika Takarada-Iemata[1]*, Takashi Tanaka[2], Loc Dinh Nguyen[1], Nahoko Okitani[1], RongRong Yan[1], Takashi Tamatani[1], Hiroshi Ishii[1], Tsuyoshi Hattori[1], Hiroyasu Kidoya[3], Yoshiaki Kubota[4], Takao Iwawaki[5], Osamu Hori[1]

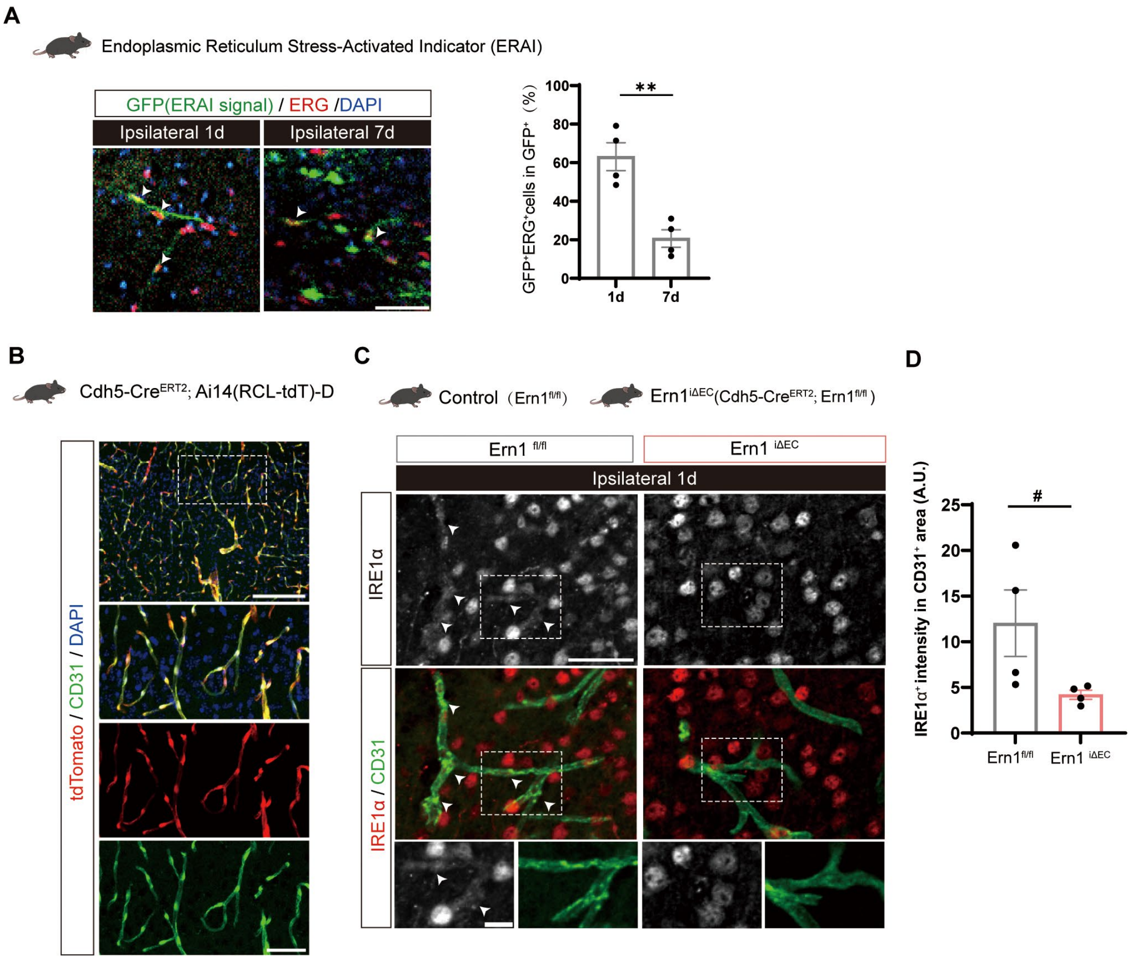

Supplementary Figure S1. Targeting IRE1 pathway in vascular endothelial cells after TBI.

(**A**) Immunofluorescence staining for GFP (green) and an endothelial nuclei marker ERG (red) in the ipsilateral cortex of ERAI mice at day 1 and day7 post-TBI. Right, percentage of GFP^+ERG^+ cells among total GFP^+ cells in the perilesional area. $**p < 0.01$ by Mann–Whitney U test (n = 4). (**B**) Representative images of CD31 (green) immunostaining tdTomato (red) in the cortex of *Cdh5-Cre^{ERT2}*; Ai14 (RCL-tdT)-D mice. Boxed region is shown at higher magnification. Scale bars: overview, 200 µm; inset, 100 µm. (**C**) Representative images of IRE1α (red) and CD31 (green) immunostaining in the ipsilateral cortex of *Ern1$^{flox/flox}$* and *Ern1$^{i\Delta EC}$* mice at day 1 post-TBI. Boxed region is shown at higher magnification. Scale bars: overview, 50 µm; inset, 15 µm. Data are presented as mean ± SEM. (**D**) Quantification of $IRE1\alpha^+$ intensity in $CD31^+$ area within a 200 µm perilesional region. $\#p < 0.05$ by Mann–Whitney U test (n = 4). Data are presented as mean ± SEM.

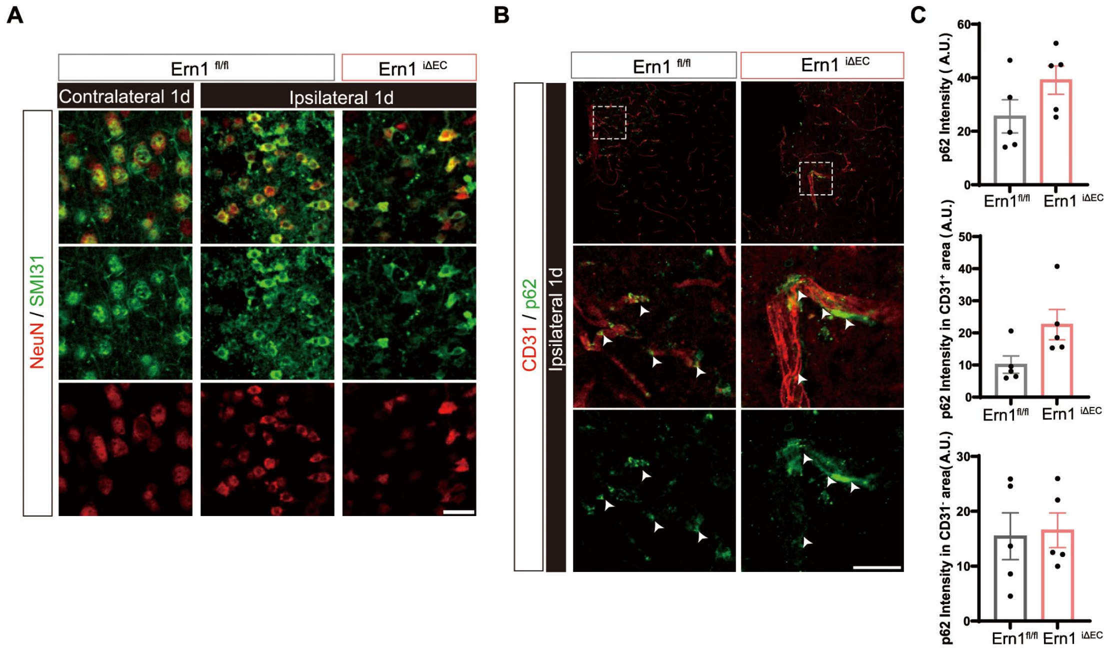

Supplementary Figure S2. Neurodegeneration and autophagy in endothelial IRE1 deficiency after TBI.

(**A**) Representative images of NeuN (red) and SMI31 (green) immunostaining in the ipsilateral cortex of *Ern1*$^{flox/flox}$ and *Ern1*$^{i\Delta EC}$ mice at day 1 post-TBI. Loss of neuron and atrophic morphological changes were observed in ipsilateral cortex after TBI. Scale bar: 20 μm. (**B**) Immunofluorescence staining for vascular endothelial marker CD31 (red) and autophagy marker p62 (green) in the ipsilateral cortex of *Ern1*$^{flox/flox}$ and *Ern1*$^{i\Delta EC}$ mice at day 1 post-TBI. Boxed region is shown at higher magnification. Arrowheads indicate p62^{+} signals in CD31^{+} vessels. Scale bar: 50 μm. (**C**) Quantification of total p62^{+}, p62^{+}CD31^{+}, and p62^{+}CD31^{-} signal intensity. n.s. by Mann–Whitney U test (n = 5). Data are presented as mean ± SEM.

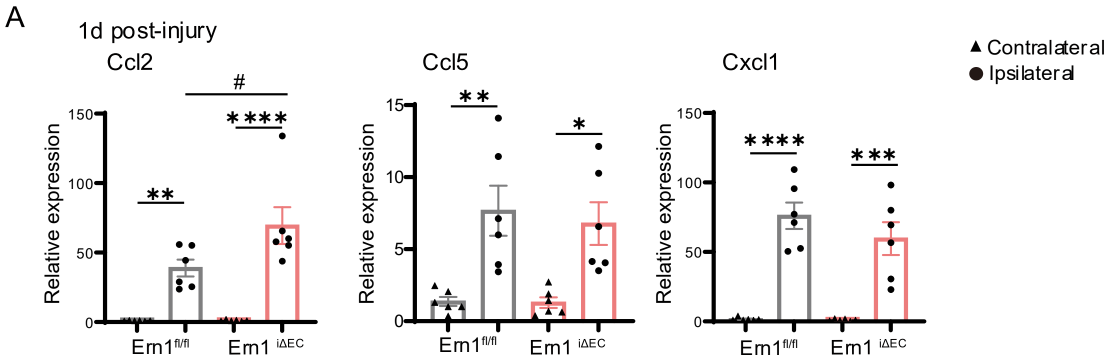

Supplementary Figure S3. Expression of chemokines in endothelial IRE1 deficiency after TBI.

(**A**) RT-qPCR analysis of *Ccl2, Ccl5,* and *Cxcl1* expression in cortices of $Ern1^{flox/flox}$ and $Ern1^{i\Delta EC}$ mice at day 1 post-TBI. *p < 0.05, **p < 0.01, ***p < 0.001, ****p < 0.0001 vs. contralateral; #p < 0.05 vs. $Ern1^{flox/flox}$ by one-way ANOVA with Tukey's test (n = 6). Data are presented as mean ± SEM.

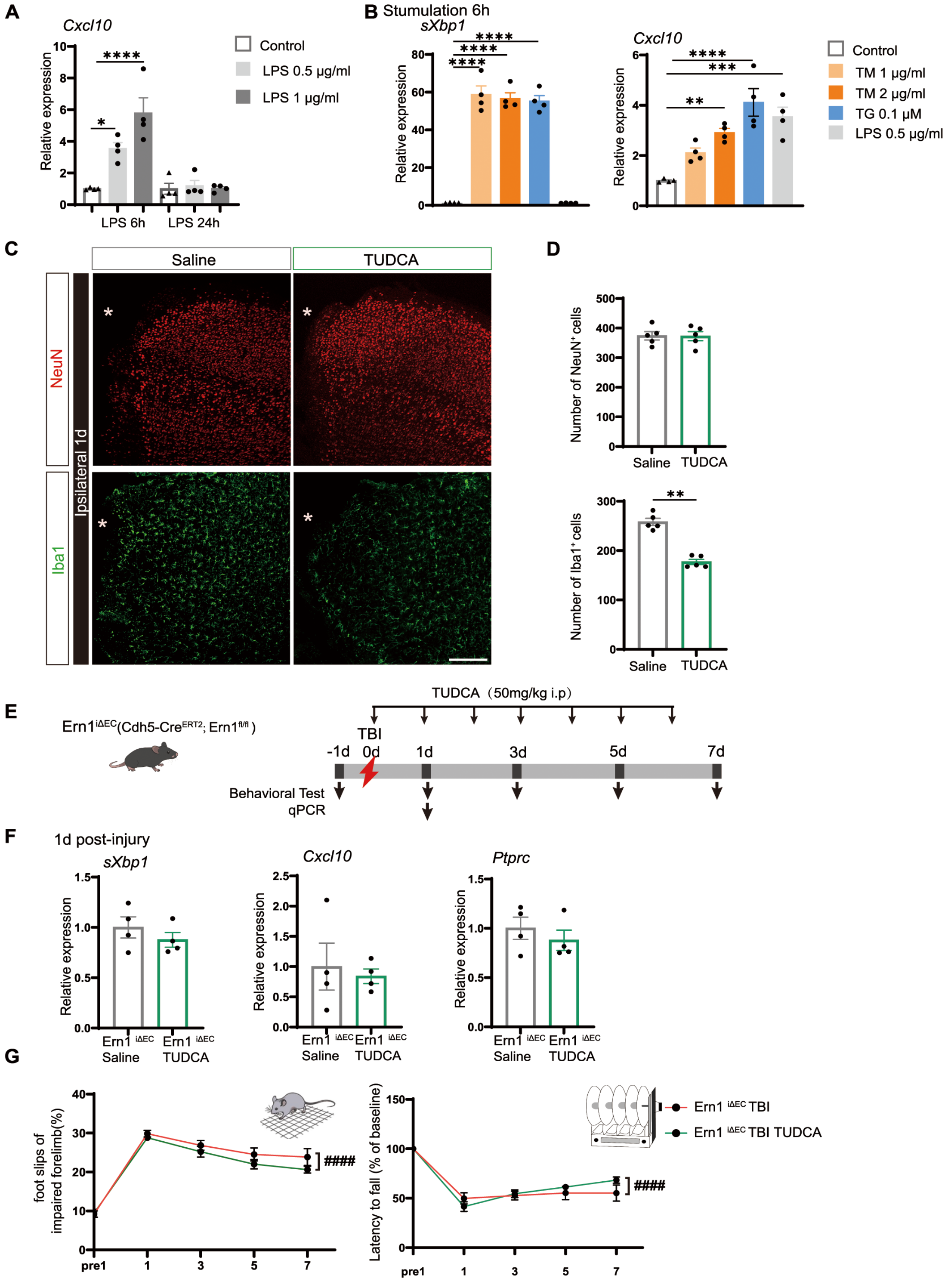
A
Cxcl10
Relative expression
LPS 6h
LPS 24h
Control
LPS 0.5 μg/ml
LPS 1 μg/ml
B Stimulation 6h
sXbp1
Cxcl10
Relative expression
TM 1 μg/ml
TM 2 μg/ml
TG 0.1 μM
LPS 0.5 μg/ml
C
Saline
TUDCA
NeuN
Iba1
Ipsilateral 1d
D
Number of NeuN+ cells
Number of Iba1+ cells
Saline
TUDCA
E
Ern1iΔEC(Cdh5-CreERT2; Ern1fl/fl)
TUDCA (50mg/kg i.p)
TBI
-1d
0d
1d
3d
5d
7d
Behavioral Test
qPCR
F
1d post-injury
sXbp1
Cxcl10
Ptprc
Relative expression
Ern1 iΔEC Saline
Ern1 iΔEC TUDCA
G
foot slips of impaired forelimb(%)
Latency to fall (% of baseline)
pre1
####
Ern1 iΔEC TBI
Ern1 iΔEC TBI TUDCA

Supplementary Figure S4. ER stressors-induced CXCL10 expression and TUDCA-mediated improved outcomes after TBI.

(**A**) RT-qPCR analysis of *Cxcl10* in bEnd.3 cells under different LPS concentrations and treatment durations. *$p < 0.05$, ****$p < 0.0001$ vs. control by one-way ANOVA with Tukey's test ($n = 4$). (**B**) RT-qPCR analysis of *sXbp1*, and *Cxcl10* in *bEnd.3* cells treated with tunicamycin, thapsigargin, or LPS at indicated concentration. **$p < 0.01$, ***$p < 0.001$, ****$p < 0.0001$ vs. control by one-way ANOVA with Tukey's test ($n = 4$). (**C**) Representative images of $NeuN^+$ neurons (red) and $Iba1^+$ microglia/macrophage (green) in the cortex of TBI-saline and TBI-TUDCA mice at day 1 post-TBI. Asterisks indicate the lesion site. Scale bar: 200 μm. (**D**) Quantification of $NeuN^+$ and $Iba1^+$ cells. **$p < 0.01$ vs. TBI-Saline by Mann–Whitney U test ($n = 5$). (**E**) Experimental timeline of TUDCA administration in $Ern1^{i\Delta EC}$ mice after TBI. (**F**) RT-qPCR analysis of *sXbp1*, *Cxcl10*, and *Ptprc* (CD45) in cortices of mice with TUDCA or saline administration at day 1 post-TBI. n.s. by Mann–Whitney U test ($n = 4$). (**E**) Grid-walk and rotarod performance over 7 days in $Ern1^{i\Delta EC}$ mice with TUDCA administration after TBI. Two-way ANOVA with Sidak's post hoc test ($n = 5$ per group). Grid-walk test (group: $p < 0.0001$, time: $p > 0.05$, group × time: $p > 0.05$). Rotarod test (group: $p < 0.0001$, time: $p > 0.05$, group × time: $p > 0.05$). Data are presented as mean ± SEM.

Supplementary Table S1. Primers used for qPCR

Gene	Forward (5’ to 3’)	Reverse (5’ to 3’)
sXbp1	GGTCTGCTGAGTCCGCAGCAGG	CTCTGGGGAAGGACATTTGA
Cxcl10	GCTGCAACTGCATCCATATC	GTGGCAATGATCTCAACACG
Ptprc	CTATCCCGCCCAGAATGGAC	TTAGCATCCTGCTTGCCTCC
Il1b	AAGCTCTCCACCTCAATGGA	AGGTGCTGATGTACCAGTTG
Tnf	CAAGCCTGTAGCCCACGTCG	ATCGGCTGGCACCACTAGTT
Cdh5	CCCGTCTTTACTCAATCCACA	ATCTGGGTCCACAACAGTCAG
Ccl2	CCAGCAAGATGATCCCAATG	TCTGGACCCATTCCTTCTTG
Ccl5	CCACTTCTTCTCTGGGTTGG	GTGCCCACGTCAAGGAGTAT
Cxcl1	ACCCAAACCGAAGTCATAGC	TGGGGACACCTTTTAGCATC
Gapdh	ACCCAGAAGACTGTGGATGG	CACATTGGGGGTAGGAACAC
