# Supplementary figures and images for: Endothelial IRE1 signaling maintains blood–brain barrier integrity and limits neuroinflammation after traumatic brain injury
Source: Cell Death Dis. 2026 Feb 9;17(1):210. doi: 10.1038/s41419-026-08461-2 (PMC12921219; doi:10.1038/s41419-026-08461-2)

# Original blots for Fig.2B

Chemiluminescence image

Brightfield image (MW markers)

IgG

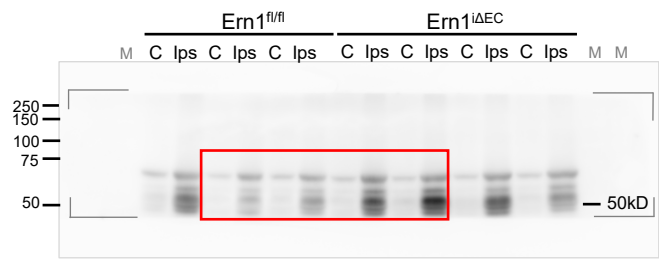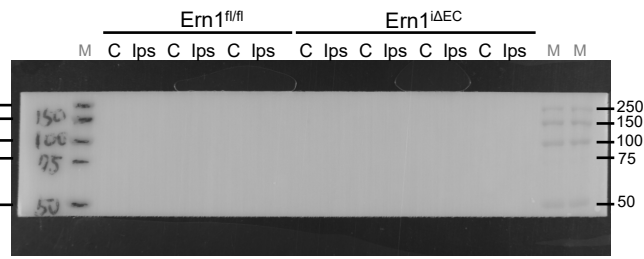

GAPDH

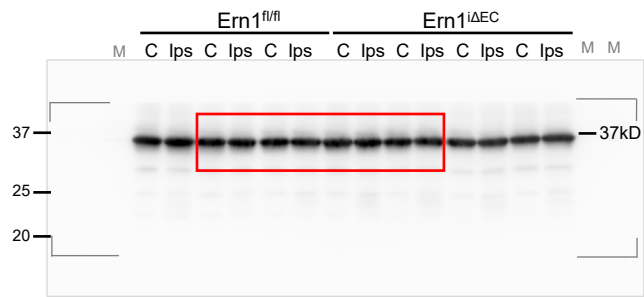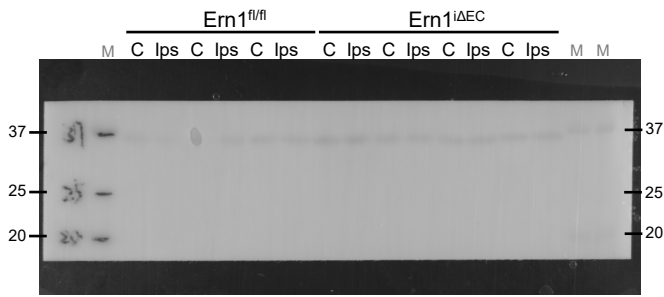

Supplement: Supplementary file 2 — Original WB data [file 41419_2026_8461_MOESM2_ESM.pdf]
